# Feasibility of a Waistband-Type Wireless Wearable Electrocardiogram Monitoring System Based on a Textile Electrode: Development and Usability Study

**DOI:** 10.2196/26469

**Published:** 2021-05-11

**Authors:** Danbi Gwon, Hakyung Cho, Hangsik Shin

**Affiliations:** 1 Department of Biomedical Engineering Chonnam National University Yeosu Republic of Korea; 2 BLACKYAK Co Ltd Seoul Republic of Korea

**Keywords:** electrocardiogram, telehealth, telemetry, telemonitoring, textile electrode, wearable system, smartphone, mobile phone

## Abstract

**Background:**

Electrocardiogram (ECG) monitoring in daily life is essential for effective management of cardiovascular disease, a leading cause of death. Wearable ECG measurement systems in the form of clothing have been proposed to replace Holter monitors used for clinical ECG monitoring; however, they have limitations in daily use because they compress the upper body and, in doing so, cause discomfort during wear.

**Objective:**

The purpose of this study was to develop a wireless wearable ECG monitoring system that includes a textile ECG electrode that can be applied to the lining of pants and can be used in the same way that existing lower clothing is worn, without compression to the upper body.

**Methods:**

A textile electrode with stretchable characteristics was fabricated by knitting a conductive yarn together with polyester-polyurethane fiber, which was then coated with silver compound; an ECG electrode was developed by placing it on an elastic band in a modified limb lead configuration. In addition, a system with analog-to-digital conversion, wireless communication, and a smartphone app was developed, allowing users to be able to check and store their own ECGs in real time. A signal processing algorithm was also developed to remove noise from the obtained signal and to calculate the heart rate. To evaluate the ECG and heart rate measurement performance of the developed module, a comparative evaluation with a commercial device was performed. ECGs were measured for 5 minutes each in standing, sitting, and lying positions; the mean absolute percentage errors of heart rates measured with both systems were then compared.

**Results:**

The system was developed in the form of a belt buckle with a size of 53 × 45 × 12 mm (width × height × depth) and a weight of 23 g. In a qualitative evaluation, it was confirmed that the P-QRS-T waveform was clearly observed in ECGs obtained with the wearable system. From the results of the heart rate estimation, the developed system could track changes in heart rate as calculated by a commercial ECG measuring device; in addition, the mean absolute percentage errors of heart rates were 1.80%, 2.84%, and 2.48% in the standing, sitting, and lying positions, respectively.

**Conclusions:**

The developed system was able to effectively measure ECG and calculate heart rate simply through being worn as existing clothing without upper body pressure. It is anticipated that general usability can be secured through further evaluation under more diverse conditions.

## Introduction

According to the World Health Organization, cardiovascular disease is the leading cause of death worldwide, accounting for 31% of all deaths [1]. Arrhythmia is a problem with the rate or rhythm of the heartbeat and is the most common and dangerous of the cardiovascular diseases. It has been reported that 15% of adults with congenital cardiovascular disease have arrhythmias, the condition occurs in 7% of patients in their 20s and 38% of patients in their 50s, and the incidence continues to increase with age [2]. Arrhythmia can cause sudden death in severe cases, and it is known that 25% of deaths from heart disease are caused by cardiac arrhythmias [3]. They are difficult to completely cure and need to be permanently managed throughout life [4]. One of the most difficult aspects of the diagnosis and treatment of arrhythmia is that it does not always persist, can occur at any point, and can disappear intermittently. Therefore, some patients are required to record their electrocardiogram (ECG) continuously for extended periods, and for this, 24-hour ECG monitoring with a Holter monitor is used clinically to accurately track and diagnose the occurrence of arrhythmias. Holter monitoring is a technology that records an ECG through standard limb leads using a small portable measuring device. A silver-silver chloride electrode is usually attached to the chest, and then an ECG is recorded by wired connection between an electrode and a necklace-type or a waist-worn–type signal acquisition device. Holter monitoring has been used for many years for precise diagnosis of arrhythmia but has the following limitations. First, self-use of Holter monitors are limited since the user’s understanding of the ECG lead is required when attaching electrodes. In addition, since recording is performed by a bulky device that needs to be placed on the chest or waist by a wired connection to the electrode on the chest, it may cause user discomfort. Furthermore, there are burdens associated with aesthetics (ie, some find them unattractive), or privacy related to an individual’s disease state may be compromised. Lastly, since Holter monitoring devices are used mainly for clinical diagnostic purposes, they are not suitable for users who want to monitor their ECG during normal daily life for the purposes of prevention, early diagnosis, and prognosis of cardiovascular diseases. Therefore, there is a growing need for novel ECG measurement technologies to expand the universe of users and to improve the discomfort associated with Holter devices. Since it is critical that the user always carries a device in order to continuously measure an ECG, wearable technology has been suggested as the most promising always-on approach. The clothing-type ECG system, which is one of several representative wearable technologies, embeds an ECG electrode and the attendant system into clothing. In general, these systems attach a conductive textile electrode made through a conductive fiber to a compressive top and connects it with a separate measuring device to obtain the physiological signal [5-16].

As an example of a clothing-type ECG system, Takahashi and Suzuki proposed a system for measuring ECGs that involved attaching conductive textile electrodes to both sleeve cuffs [17]. In another example, a conductive fiber was embroidered at the position of an ECG electrode on a top, and the signal was then recorded by connecting embroidered conductive yarn to a measurement system [18]. In this approach, a conductive fiber was also embroidered between the textile electrode and the system and used as an electrical wire. In addition, there is a method for measuring the user’s ECG in a noncontact manner by attaching a capacitive ECG electrode to the outside of clothing [19]. As described, the clothing-type ECG systems do not need a sensor attachment to the body but are simply integrated with the clothing to be worn in the same manner, thus improving user convenience. However, in terms of practical use, these systems have the following limitations. First, a textile electrode used in a clothing-type system has a lower impedance and lower contact stability than a medical grade electrode, and this may degrade the quality of the signal to be measured. In addition, since the electrode is generally located in or around a specific part of the upper body that experiences a lot of movement (eg, heart and upper limbs), it is vulnerable to motion artifacts.

In order to solve these problems, a method of improving the contact force of the sensor by using clothing in a form that is highly elastic and compressive to the body has been proposed, but the limitation with this approach is the significant discomfort caused by sustained compression of the user’s body. Therefore, a compelling need exists to develop a new ECG measurement technology to solve some of the problems of the existing clothing-type wearable systems.

This study aimed to develop a wireless wearable ECG monitoring system that included a textile ECG electrode that can be applied to the lining of pants and can be used in the same way as existing clothing wearing styles without upper body compression. Since the electrode is in close contact with the skin through the usual manner of wearing lower garments—tightening at the waist to make the clothes ride at the waist or pelvis—the contact force between the electrode and the skin can be improved without pressure to the upper torso with the conventional clothing-type ECG method. In addition, there is the advantage of being able to perform this monitoring simply through the normal daily activity of dressing without additional manipulations to attach electrodes. The measurement system we have developed is in the form of a belt buckle, and users can check their ECG as needed through a smartphone app.

## Methods

### Modified Limb Leads

**Figure 1:**
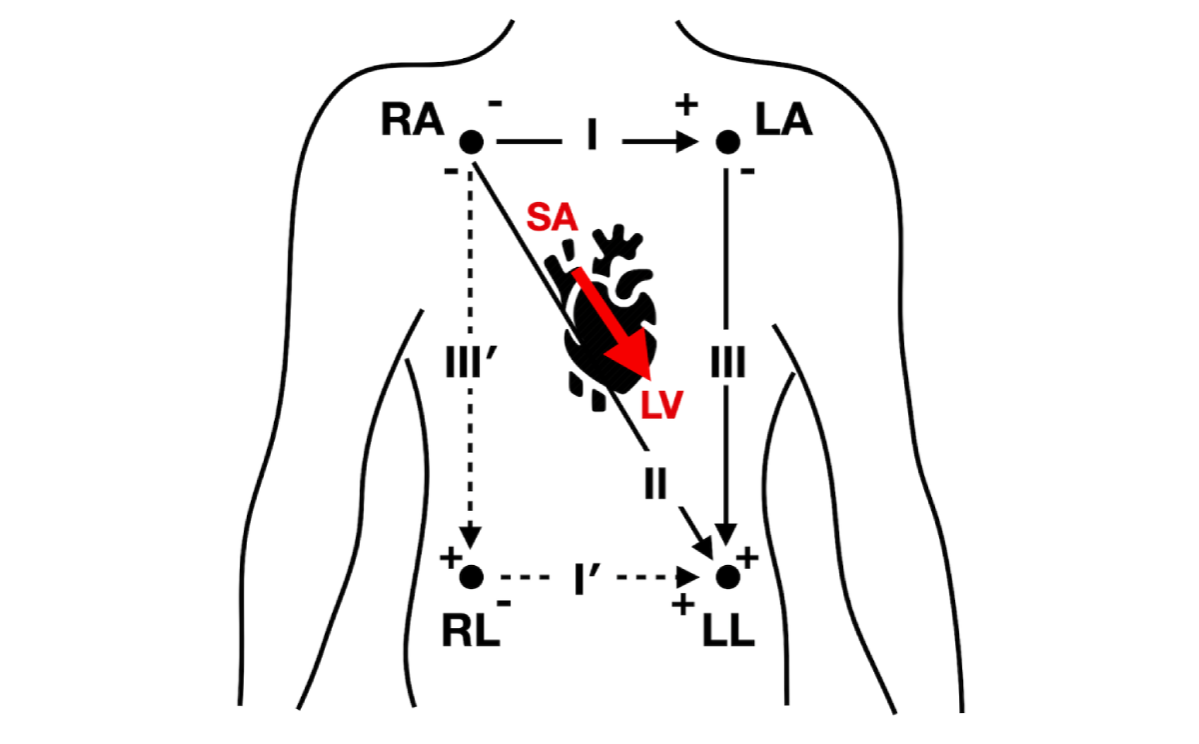
Modified limb leads (I, II, and III). LA: left arm; LL: left leg; LV: left ventricle; RA: right arm; RL: right leg; SA: sinoatrial.

An ECG measures the electrical activity of the heart on the surface of the human body and refers it to an electrical vector generated by the heart. The most representative method for performing an ECG measurement is by use of the standard limb leads developed by Einthoven. In the standard limb leads system, a total of four electrodes are attached to the arms and legs by crossing the heart, and an ECG is induced from the potential difference between the electrodes. The ECG can be measured three different ways based on the reference position for obtaining the potential difference. The ECG is recorded from the potential difference between the right and left arm for lead I, between the right and left leg for lead II, and between the left arm and left leg for lead III. The rationale behind this lead system is that bioelectricity in the heart is propagated from the right upper limb to the left lower limb. Intracardiac electrical conduction begins at the sinoatrial (SA) node in the upper wall of the right atrium, passes through the atrioventricular (AV) node located between the atrium and the ventricle, passes through this bundle, and is delivered to Purkinje fibers, a type of fine nerve fiber distributed in the ventricle. Therefore, the directionality of electrical conduction of the heart occurs from the head to the foot; in addition, since the ventricular wall of the left ventricle responsible for systemic circulation is thicker and the distribution of Purkinje fibers is higher, the electrical conduction vector is skewed to the left rather than the right. As a result, electrical conduction of the entire heart occurs from the SA node to the left ventricle, which corresponds to the direction from the right arm to the left leg. Figure 1 shows the direction of electrical conduction and the ECG lead system. The solid diagonal line in Figure 1 shows the standard limb lead of Einthoven’s triangle. Lead II is the most similar to the electrical conduction direction of the heart, and it is here that the ECG with the largest amplitude is observed. Lead II can be expressed as a vector sum of horizontal and vertical directions, and these can be defined as leads I and III, respectively.

The standard limb leads should have at least one electrode attached to an upper limb for measurement; thus, conventional clothing-type wearable devices have been mainly developed in a form that has electrodes attached to the top. However, attaching the electrode to the upper body may cause discomfort due to upper limb compression. To solve this problem, we decided to move all electrodes to the bottom, and for this we used a form of modified limb leads. In Figure 1, the vector corresponding to lead II can also be expressed as the sum of vectors I' and III'; in this case, the potential difference between both lower limbs can be measured through lead I'. This lead system does not pressurize or attach an additional electrode to the upper body, so it does not restrict activity and provides the same user experience as wearing items of clothing like pants. As such, it is more convenient than existing wearable ECG systems that attach an electrode on the upper limb. This type of lead system has been previously defined, and the leads are termed “modified limb leads” in the studies of John and Sivaraman [20] and Sivaraman et al [21]. In their studies, they report that the modified limb lead is more useful for observing ECG amplitude or frontal plane axis shift than standard limb leads.

### Waistband-Type ECG Electrodes

#### Fabrication Process for the Textile Electrode

**Figure 2:**
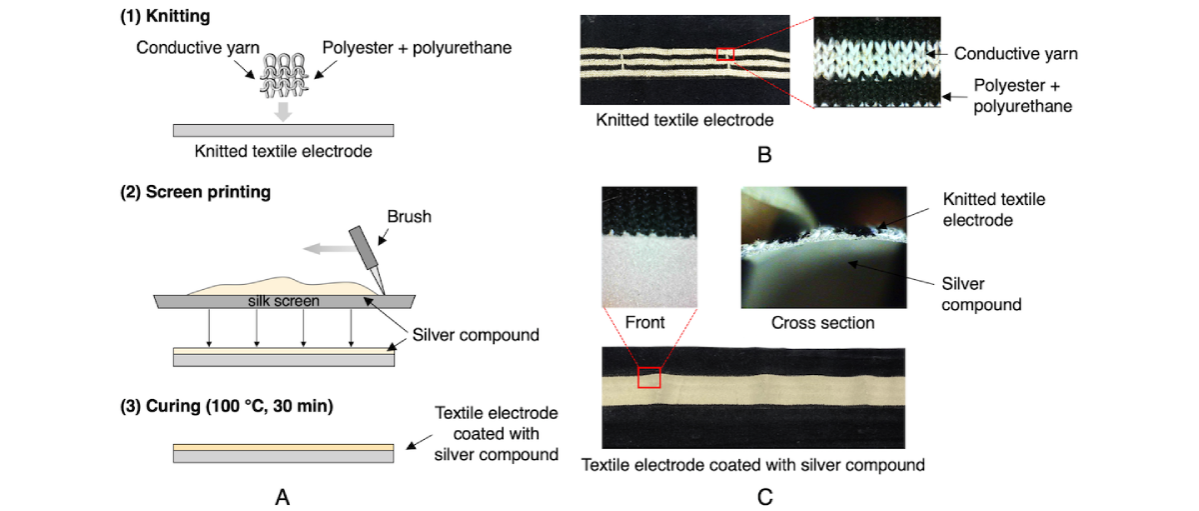
Fabrication process and configuration of the knitted textile electrode for electrocardiogram measurement. A. Fabrication process of the knitted textile electrode. B. Structure of the knitted textile. C. Knitted textile electrode coated with silver compound.

To develop the textile electrode, conductive fibers were made by covering a 70-denier polyester core fiber with two strands of 30-μm diameter silver fibers. In order to obtain elasticity and a close fit of the electrode, a textile electrode knitted with conductive fiber possessing a striped structure was prepared from 18% polyurethane, 82% polyester, and the conductive fiber. For better contact with the skin and more accurate measurement, the knitted textile electrode was coated with a silver compound. The silver compound was selectively printed on the conductive fibers with a silk screen then cured at 100 ℃ for 30 minutes. Silver coating was limited to the area of the knitted fabric containing the conductive fibers, based on considerations of elasticity, conductivity, close contact, possible irritation, and sewing productivity (Figure 2, A). Panels B and C in Figure 2 show the structure of the knitted textile and the knitted textile electrode coated with silver compound, respectively. The silver compound used in this study is a conductive resin, XX-ECA05 (CEMEDINE Co Ltd), which is composed of elastomeric resin from acrylic resin, diethyl ether, and ethylene glycol derivatives as well as silver and its water-soluble compounds. The composition and properties of XX-ECA05 are shown in Table 1.

**Table 1:** Ingredients and material properties of the conductive resin XX-ECA05 (CEMEDINE Co Ltd).

Ingredient name or property			Content, %		Description	
**Ingredient**						
	Acrylic resin	10-20		N/A^a^		
	Diethyl ether	1-10		N/A		
	Ethylene glycol derivatives	1-5		N/A		
	Silver and its water-soluble compounds	69		N/A		
**Material property**						
	Usage	N/A		Printable, stretchable wiring		
	Base resin	N/A		Elastomer		
	Viscosity	N/A		60.0 Pa·s		
	Curing condition	N/A		100 ℃ × 30 min		
	Working temperature	N/A		–20 ℃ to 80 ℃		
	Volume resistivity	N/A		0.45 mΩ·cm		
	Elongation	N/A		100%		

^a^N/A: not applicable; content was only determined for ingredients and descriptions were only provided for properties.

#### Electrode Configuration on Waistband

**Figure 3:**
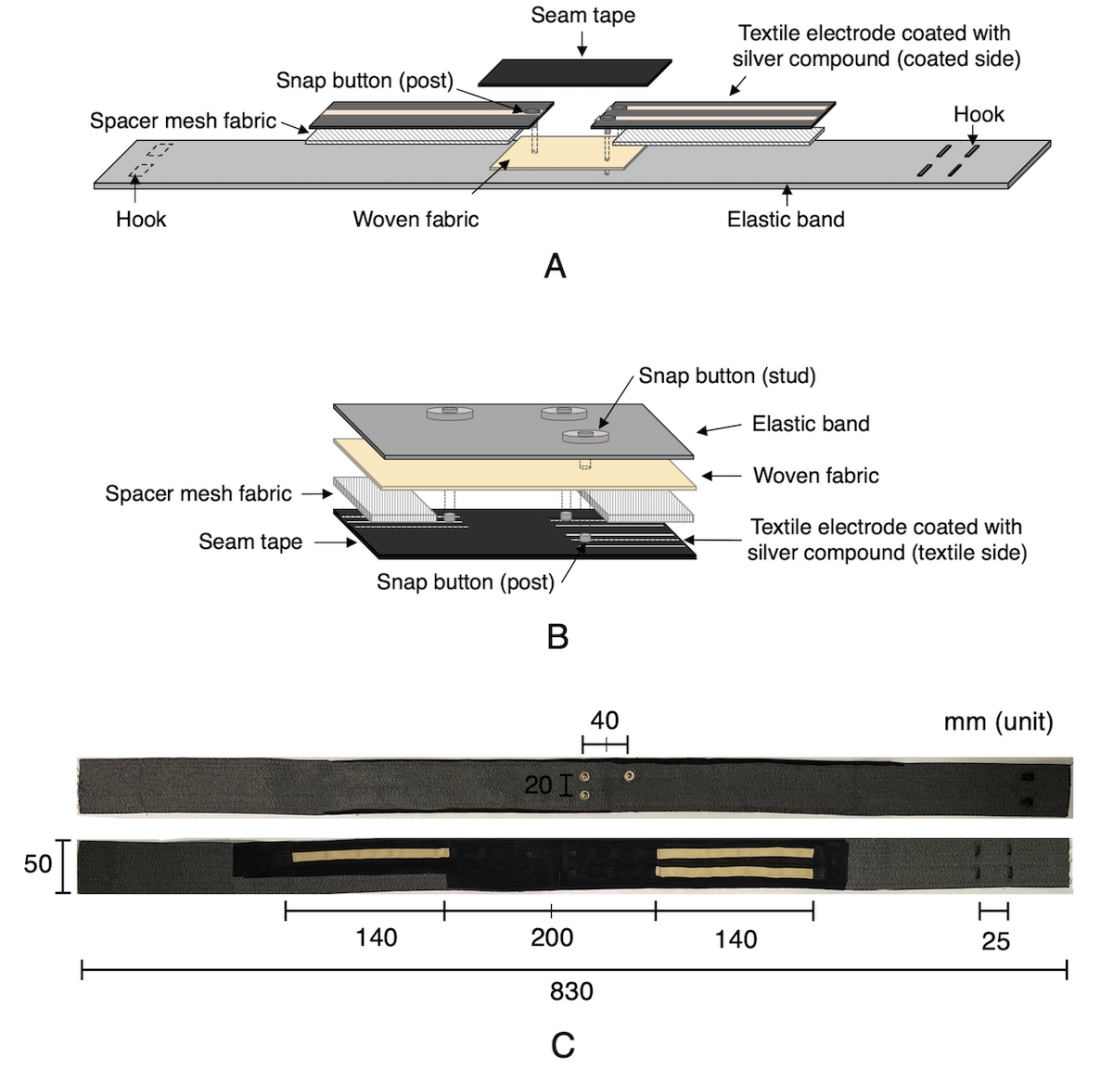
Waistband-type electrocardiogram (ECG) lead system using knitted textile electrodes. A. Configuration diagram of waistband for ECG measurement. B. Configuration diagram of snap button for connection between knitted textile electrode and ECG measurement device. C. Appearance of manufactured waistband-type ECG lead system.

In order to fit various sizes and shapes of potential users, a waistband-type ECG lead system was proposed based on an elastic band and the knitted textile electrode. The knitted textile electrode was positioned on the elastic band according to the modified standard limb lead I. An elastic mesh fabric spacer was placed between the elastic band and the knitted textile electrode, thus allowing better contact with the skin [7]. The electrode and the signal acquisition device were connected using a nickel snap button. To ensure that the metallic surface of the snap button would not directly touch the skin, the back surface was covered with seam tape. To prevent deformation of the waistband by the snap button where it connects to the measuring device, a nonelastic woven fabric was placed between the knitted textile electrode and the elastic band (Figure 3, A and B). The length of the elastic band was 830 mm; this was based on the 2015 Size Korea survey, which revealed the average waist size of 25-to-29-year-old subjects was 830.1 (SD 88.5) mm [22]. A two-step size adjustment was made possible by attaching size-adjusting hooks 25 mm apart. The size of the developed waistband was 830 mm long and 50 mm wide; the knitted textile electrodes that were cut to a length of 220 mm were symmetrically placed 20 mm apart from the horizontal center line in the front of the clothing construction to connect with devices. The snap button was positioned 40 mm away horizontally and 20 mm away vertically from the center front line. Thus, each electrode was exposed starting 100 mm from the center line, and an electrode length of 140 mm was in contact with the skin (Figure 3, C).

### Wireless ECG Measurement System

The wearable ECG system was connected to a waistband-type electrode through a snap button, measured the ECG, and transmitted it wirelessly to an Android smartphone. The app displayed the received ECG as a real-time graph and stored it as a file. The data acquisition system consisted of an ADS1191 (Texas Instruments Inc), which is a commercial, analog front end for ECG measurement; an Arduino Pro Mini (SparkFun Electronics), which is an Atmega328-based microcontroller unit (MCU); and an HC-06 Bluetooth communication module (Guangzhou HC Information Technology Co Ltd). The ADS1191 is a 1-channel analog front end that amplifies the ECG and converts the analog signal to digital. The common mode rejection ratio of the ADS1191 is –95 dB and it supports a sampling frequency of 125 to 8000 samples per second with 16-bit analog-to-digital conversion resolution. The Arduino Pro Mini is a microcontroller that operates at up to 16 MHz and supports 14 digital inputs and four analog inputs. The size of the Arduino Pro Mini, at 18 × 33 mm, is suitable for developing small systems. The HC-06 Bluetooth module supports Bluetooth version 2.0 serial port profile and transmits ECG data received from the MCU to the smartphone. Serial peripheral interface communication between the ADS1191 and the Arduino Pro Mini, as well as universal asynchronous receiver/transmitter communication between them, are used for data communication. Table 2 summarizes the system specifications. The buckle-type system cover was designed using 123D Design, version 2.2.14 (Autodesk), a freeware computer-aided design software application, and was printed using the Replicator2 3D printer (MakerBot). The polylactic acid filament, a transparent plastic material, was used to make the cover. The Android app for ECG self-monitoring was developed to run on the SM-N920K smartphone (Samsung Electronics, Inc) based on Android Software Development Kit 24 using Android Studio, version 3.5.3. After receiving data from the digital system’s Bluetooth module, the app would display the ECG waveform in real time and store the received ECG data as a comma-separated value format file to the device storage space. The MPAndroidChart library, a graph generation library, was used to display real-time ECG waveforms [23].

**Table 2:** Specifications of the developed system.

Module and specification		Value	
**Analog front end (ADS1191)**			
	Analog-to-digital conversion resolution	16 bits	
	Sampling rate	125 samples per second	
**Microcontroller (Arduino Pro Mini)**			
	Clock speed	16 MHz	
	Operating voltage	3.3 V	
**Bluetooth module (HC-06)**			
	Bluetooth version	V2.0	
	Operating voltage	3.2-6 V	
	Operating frequency	2.4 GHz	
**Total system, including cover and battery**			
	Size (width × height × depth)	53 × 45 × 12 mm	
	Weight	23 g	

### Signal Processing Algorithm for Heart Rate Estimation

**Figure 4:**
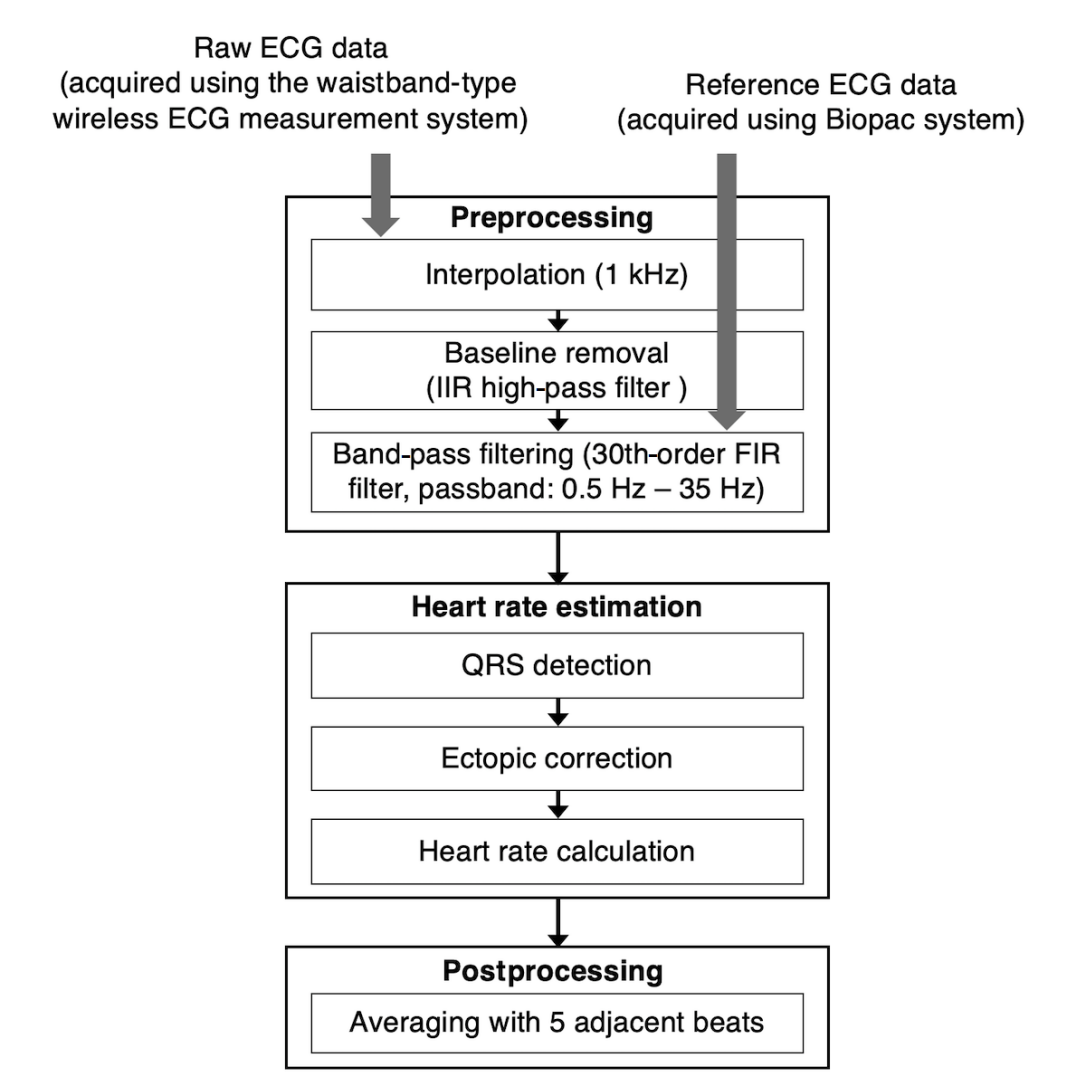
Algorithm for preprocessing electrocardiogram (ECG) and estimating heart rate. FIR: finite impulse response; IIR: infinite impulse response.

The waistband-type ECG system does not use adhesive foam for attachment to the human body, and the electrode-skin contact state may vary drastically by movement, so compared to existing ECG systems, it is quite vulnerable to motion artifacts, including noise from respiratory movement. In addition, data may be lost due to communication delay or packet loss during wireless data transmission. Therefore, in order to accurately extract the QRS complex and estimate heart rate, a signal processing procedure for noise removal and signal restoration is required. Figure 4 shows the algorithm for extracting heart rate from the waistband-measured ECG. First, since a transmission delay or loss may occur in the process of receiving a signal, the obtained signal was interpolated at 1 kHz to compensate for uneven spacing of the signal. Then, an infinite impulse response high-pass filter was applied to reduce baseline fluctuation. Thereafter, a 30^th^-order finite impulse response filter having a 0.5 Hz to 35 Hz passband known as a general ECG frequency band was applied. After the preprocessing stage, QRS complexes were detected using the Pan-Tompkins algorithm [24], then the RR interval (RRI) was calculated based on the detected QRS. At this time, a QRS detection error may occur due to an incorrect QRS detection or transmission error, and this may cause a phenomenon in which the RRI has an out-of-range value that is abnormal. This can lead to a fatal error in calculating the heart rate. Therefore, after calculation of the RRI, abnormal range value correction is required. To this end, the RRI was reconstructed using the algorithm proposed by Morelli et al [25]. In this algorithm, the number of lost beats is estimated from the abnormal value; from this, the average RRI value and the lost beats are estimated and restored. After correction, the heart rate is calculated by taking its reciprocal. When the heart rate is calculated for each RRI, it may appear that it changes very rapidly due to a subtle error in the QRS position. For example, when calculating the heart rate with only a one-beat interval, if the QRS position is measured outside of 50 milliseconds due to a detection error, an error of about 5 beats per minute (bpm) may occur based on a heart rate of 72 bpm. Therefore, in order to reduce the influence of these minute fluctuations, it is common to obtain several adjacent beats and provide an average beat rate. In this study, the average bpm was calculated from the average of 5 beats, and for this, a 5-point moving average filter (MAF) was applied.

### Validation

To measure the surface resistance of the silver compound–coated electrode, an EC-80P (NAPSON KOREA) surface resistance meter was used. Surface resistance was measured for samples either treated or not treated with silver compound coating. In the case of coated samples, surface resistance was measured for each of the coated and back surfaces. When measuring sheet resistance, a load of 68 Pa was applied to the textile electrode to ensure contact was made with the probe of the sheet resistance meter. Since the result may vary for each measurement, three measuring locations were randomly selected for each sample, and then the average surface resistance was calculated by repeating measurements for each location 10 times.

To evaluate the heart rate measurement performance of the waistband-wearable device, it was simultaneously compared to a commercial system used to obtain ECGs, and then heart rate estimations were compared and evaluated. The MP150 and ECG100C systems (Biopac Systems Co Ltd) were used for obtaining reference ECGs, and these were obtained through lead I by attaching electrodes under the right and left clavicle bones. The waistband-type ECG system was worn naturally under the navel, and signals were recorded simultaneously with the MP150. Acknowledge 4.3 was used to display and store ECGs obtained from the MP150 on a computer, and the in-house smartphone app was used to store data obtained from the waistband ECG system. Signals were measured for at least 5 minutes each in the standing, sitting, and prone positions to evaluate measurement performance during various postures. The ECG signal was sampled at 1 kHz for the commercial systems and 125 Hz for the manufactured systems. This study was approved by the institutional review board (IRB) of Chonnam National University, South Korea (IRB No.1040198-200609-HR-061-02). The correspondence between heart rates measured by the reference ECG system and the developed system were investigated using differences in the mean and standard deviation, the mean absolute error (MAE), and the mean absolute percentage error (MAPE) and by Bland-Altman analysis. Comparisons between heart rates were qualitatively evaluated.

## Results

### Textile Electrode

**Figure 5:**
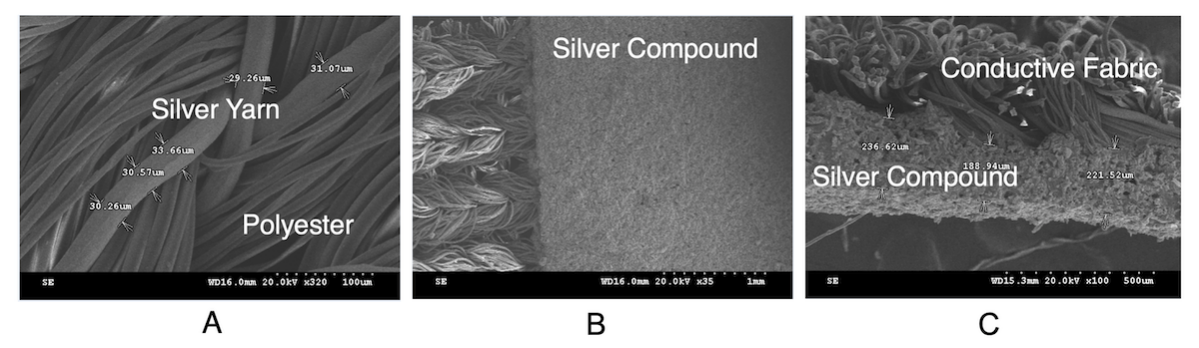
Scanning electron microscope images of textile electrode. A. Knitted textile electrodes without silver compound coating (320× magnification). B. Knitted textile electrodes with silver compound coating (35× magnification). C. Cross-section of knitted textile electrodes with silver compound (100× magnification).

Figure 5 shows scanning electron microscope images of the fabricated textile electrode. The panels show the surface of the knitted textile electrode before silver compound coating (Figure 5, A), the surface of the knitted textile electrode after silver compound coating (Figure 5, B), and the side view cross-section (Figure 5, C). In Figure 5, A, it was shown that the knitted textile electrode was composed of silver yarn of about 30 μm in diameter with several strands of polyester. Panels B and C of Figure 5 demonstrated that the silver compound was applied with a thickness of about 190 to 240 μm. Measurement of the average surface resistance of silver-coated and uncoated electrodes revealed the surface resistance of the sample without silver coating to be 202.0 (SD 43.1) Ω/sq, whereas the surface resistance of the silver-coated and back sides were 0.22 (SD 0.03 Ω/sq) and 0.17 (SD 0.02 Ω/sq), respectively. These results demonstrated that surface resistance was dramatically reduced by the silver compound coating.

### Wireless ECG Measurement System

**Figure 6:**
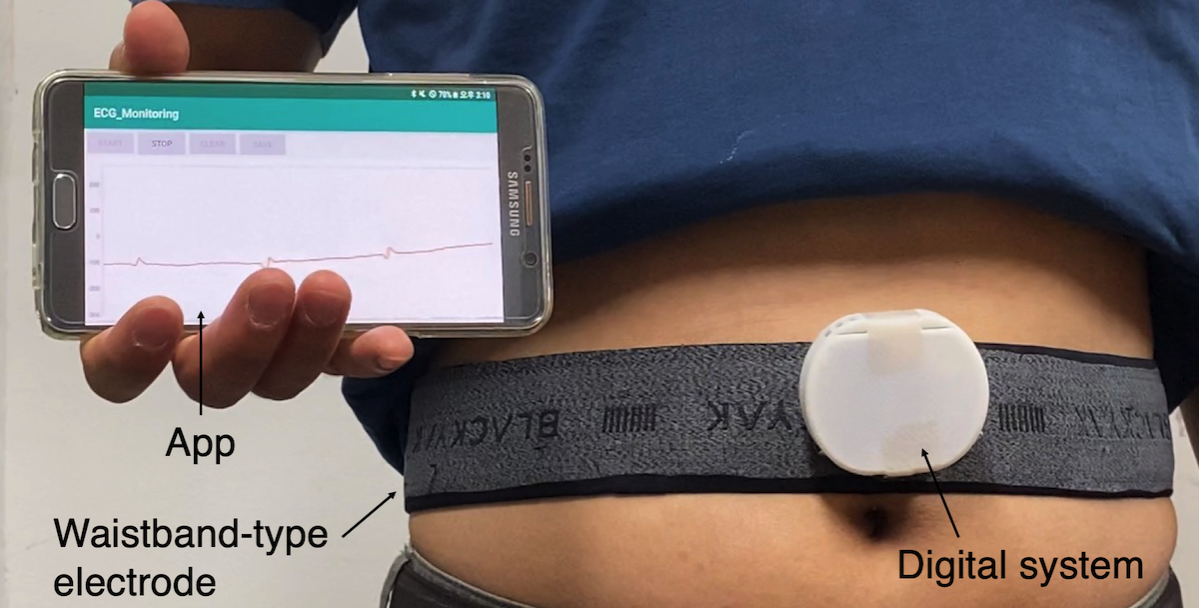
Developed waistband-type wearable electrocardiogram (ECG) monitoring system, including waistband-type ECG electrode, acquisition system, and smartphone app.

The size of the developed system was 53 × 45 × 12 mm (width × height × depth, including the cover) and it weighed 23 g. Figure 6 shows that the system based on our waistband-type wearable ECG electrode can measure an ECG. From the ECG waveform obtained, the QRS complex can be identified. Figure 6 displays the system components and shows that the measured ECG was successfully transmitted to a smartphone in real time. Feedback from study subjects with regard to the comfort of wearing a waistband-type wearable device elicited responses typified by “the same fit as normal pants” and “no awareness of wearing a separate sensor.”

### Heart Rate Estimation

**Figure 7:**
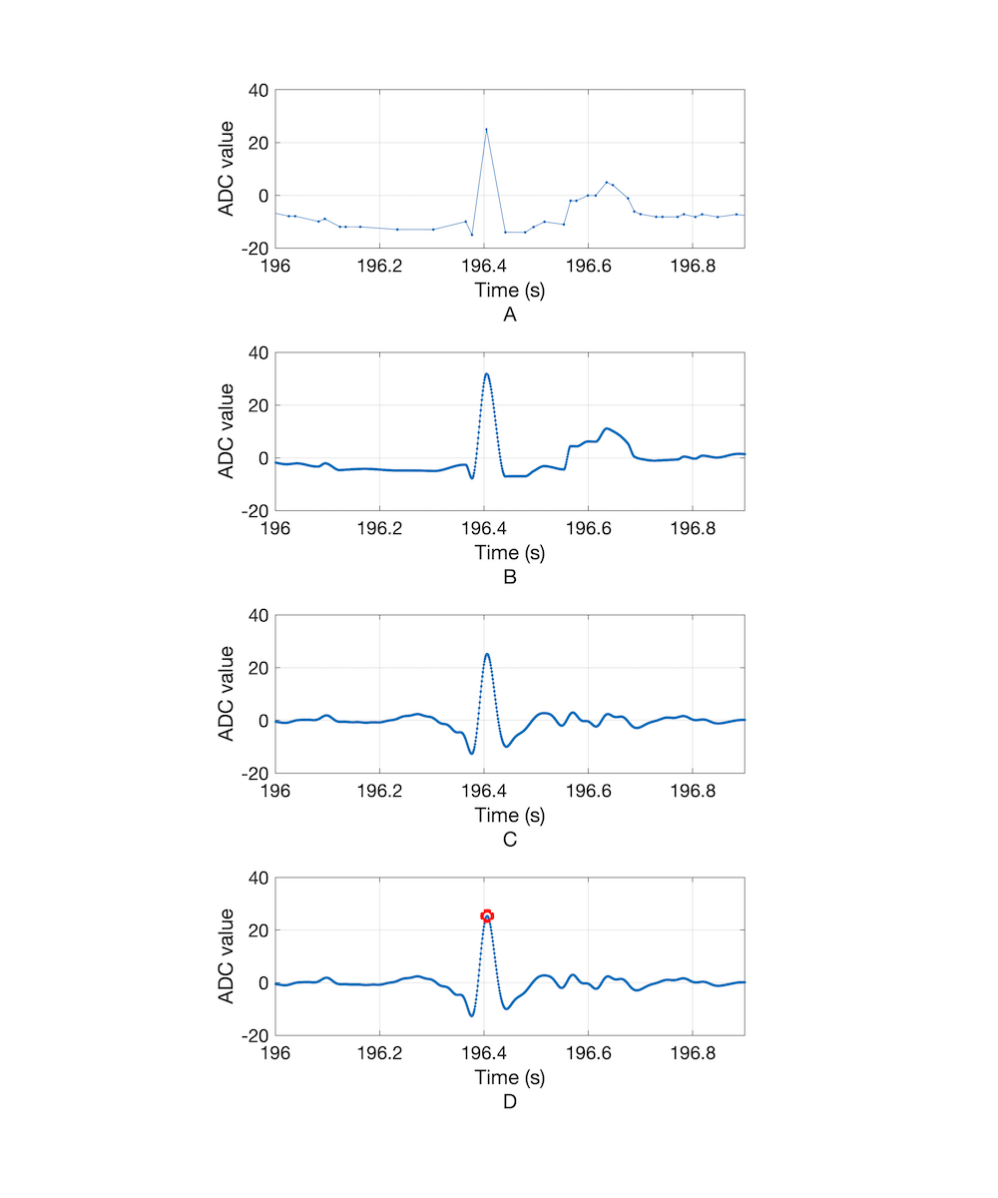
Electrocardiogram waveform modified by signal processing procedure. A. Signal obtained via Bluetooth communication. B. Signal interpolated with 1-kHz sampling frequency. C. Signal filtered with 0.5-35-Hz finite impulse response band-pass filter. D. Detected QRS complex (red circle). ADC: analog-to-digital conversion.

**Figure 8:**
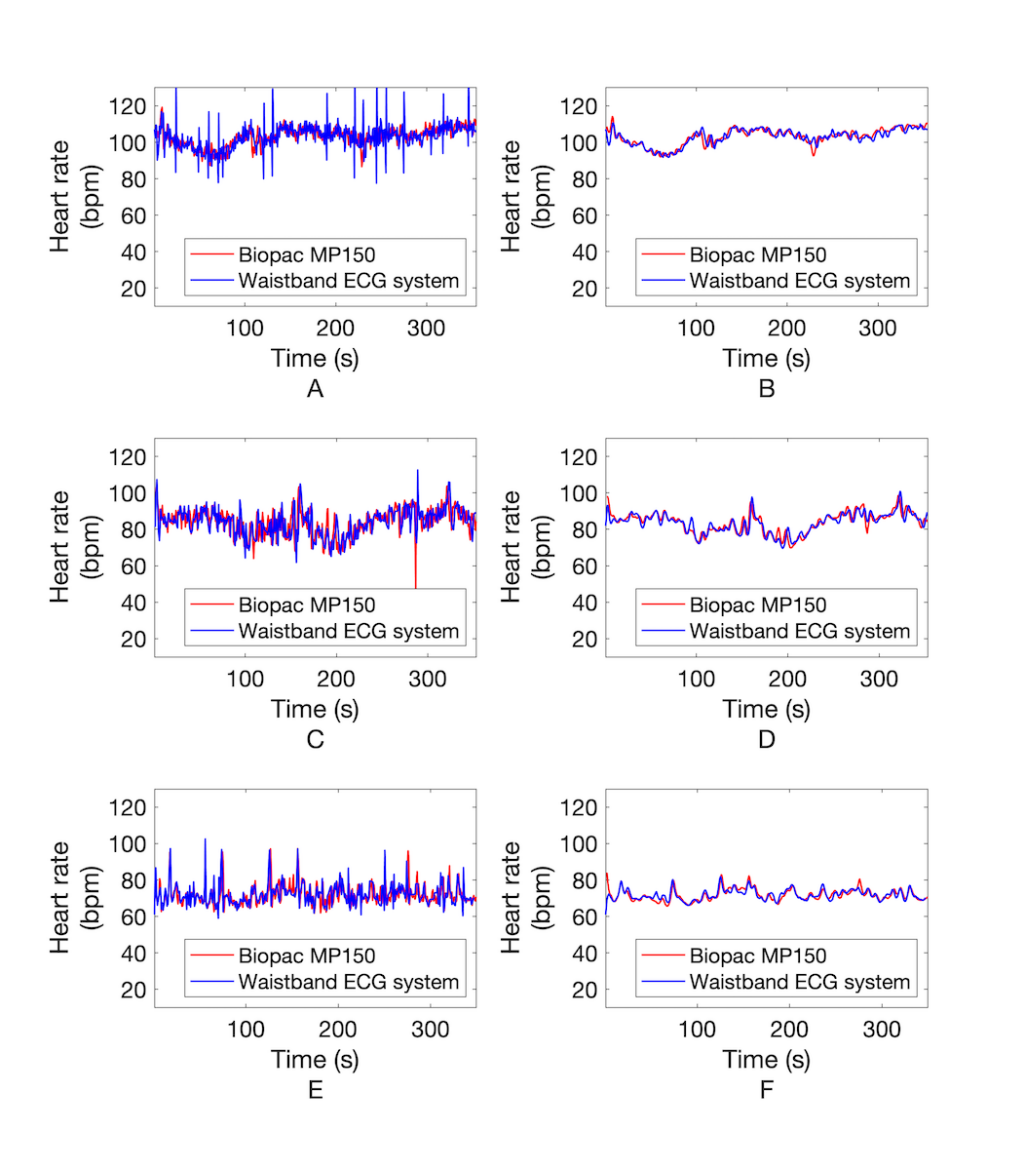
Bland-Altman and Pearson correlation plots. Bland-Altman plot (A) and Pearson correlation plot (B) in standing position. Bland-Altman plot (C) and Pearson correlation plot (D) in sitting position. Bland-Altman plot (E) and Pearson correlation plot (F) in lying position. bpm: beats per minute; ECG: electrocardiogram.

**Figure 9:**
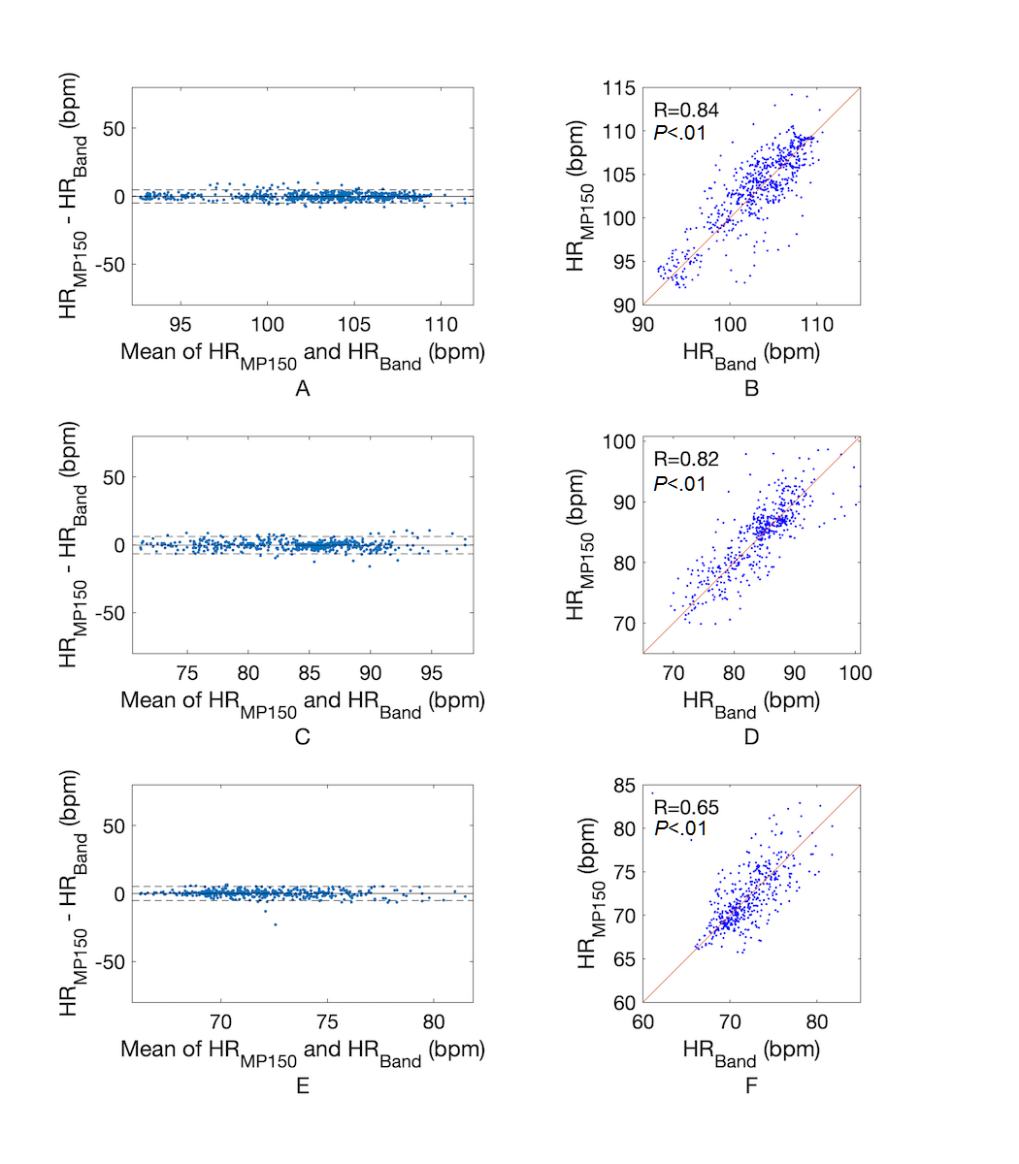
Heart rate change by applying the moving average filter (MAF). A. In standing position without MAF. B. In standing position with MAF. C. In sitting position without MAF. D. In sitting position with MAF. E. In supine position without MAF. F. In supine position with MAF. bpm: beats per minute; HR: heart rate; R: Pearson correlation coefficient.

Figure 7 shows an example of changes to the ECG waveform based on signal processing for heart rate estimation. Figure 7, A, shows the signal received from the waistband system, in which the data interval is uneven due to deviations in the data transmission time with Bluetooth communication. Figure 7, B, shows that the ECG waveform after 1-kHz interpolation becomes uniform in interval. Figure 7, C, shows the ECG after 0.5-to-35-Hz band-pass filtering, and here the QRS waveform becomes clearer after filtering. Figure 7, D, shows the QRS complex detected by the Pan-Tompkins QRS detection algorithm (red circle). In some cases, the RRI calculated from beat loss or waveform distortion may have a value far outside the normal heartbeat interval range (eg, >1500 milliseconds). However, after correction, all the RRIs in the abnormal range disappeared and were corrected to values ​​within the normal range. Figure 8 shows the results of heart rate estimation before and after applying the MAF. Panels A, C, and E of Figure 8 are heart rate changes in the standing, sitting, and supine positions, respectively, before using the MAF; these plots show very rapid changes, such as high-frequency noise caused by minute errors in beat intervals. On the other hand, panels B, D, and F of Figure 8 are heart rate changes after the MAF was applied in the standing, sitting, and supine positions, respectively; these plots have a trend similar to that seen when the heart rate changes gradually over time. Also seen in Figure 8, the red line is the heart rate change measured by the standard ECG measuring device MP150, and the blue line is the heart rate change measured by the developed device. From this result, it can be qualitatively confirmed that the developed device tracks the heart rate change accurately. The mean error, MAE, and MAPE of heart rates calculated from the ECG signal obtained through the developed system and the reference ECG signal are shown in Table 3. From this, it can be seen that the developed system has an MAE of 1.85 bpm in the standing position, 2.39 bpm in the sitting position, and 1.81 bpm in the lying position compared to the reference system. MAPEs were 1.80%, 2.84%, and 2.48% in the standing, sitting, and lying positions, respectively, which is acceptable and within the 10% validity criterion used in a recent study [26], as well as within the 5% validity criterion, which has been strictly applied [27]. Bland-Altman analysis (Figure 9 and Table 3) revealed that most of the mean differences between heart rates measured by both systems were within limits of agreement (LoA), as represented by dashed lines in the figure. LoA analysis revealed that for 95% of cases, the heart rate measured by the developed system will be from 0.95 to 1.04, from 0.92 to 1.07, and from 0.93 to 1.07 times the reference system in the standing, sitting, and lying positions, respectively. This indicates that heart rates measured by the developed system may differ from the reference system heart rate by 5% to 8% below to 4% to 7% above. In addition, Pearson correlation coefficients between the reference and developed systems were 0.84, 0.82, and 0.65 in the standing, sitting, and lying conditions, respectively.

**Table 3:** Heart rate descriptive statistics, device error statistics, and Bland-Altman analyses comparing Biopac MP150 and waistband-type wireless electrocardiogram measurement systems by condition.

Position and device			Observations, n		Heart rate (bpm^a^), mean (SD)		Device error							Bland-Altman analysis, LoA^b^		
							MAE^c^		MAPE^d^, %		Mean error (SD)		Lower			Upper
**Standing**																
	Biopac MP150	607		103.3 (4.5)		1.85		1.80		0.23 (2.47)		–5.08			4.61	
	Waistband	607		103.1 (4.3)												
**Sitting**																
	Biopac MP150	491		84.2 (5.6)		2.39		2.84		0.25 (3.27)		–6.66			6.16	
	Waistband	491		84.0 (5.4)												
**Supine**																
	Biopac MP150	416		72.0 (3.3)		1.81		2.48		–0.03 (2.65)		–5.16			5.22	
	Waistband	416		72.0 (3.0)												

^a^bpm: beats per minute.

^b^LoA: limits of agreement.

^c^MAE: mean absolute error.

^d^MAPE: mean absolute percentage error.

## Discussion

### Principal Findings

In this study, a novel ECG device based on a waistband-type electrode and wireless ECG system was developed to avoid some of the disadvantages of conventional wearable devices, and its performance was verified by comparing it with a commercial device. The textile electrode was fabricated with a structure in which conductive and elastic yarns, such as polyurethane, were knitted together to provide both conductive and stretchable characteristics. The results show that this approach allowed development of an electrode that can measure an ECG and has a degree of extensibility that does not cause inconvenience during use. In addition, the impedance of the electrode was significantly reduced through silver compound coating. For ECG measurement and heart rate monitoring, representative features of the electrical signal were observed, and the MAPE of the developed system was less than 3% in heart rate measurement. This is better than the MAPEs of commercially available heart rate monitors, such as the Apple Watch or Fitbit, as reported in a previous study [26]. These results suggest that the developed system can be reliably used to obtain an ECG and to monitor heart rate. However, in real-time transmission and recording, transmission delay or loss may occur depending on the state of the communication channel. Therefore, in the future, there is a need to secure a more reliable wireless communication channel and an error correction technology for optimization. In terms of wearer comfort, the developed device has shown the possibility that such designs can be user friendly and not cause discomfort to the user. However, factors such as fit and comfort may eventually vary depending on the packaging and design of the device and, thus, need to be verified through usability evaluation after development of the final versions.

Despite the results of the study showing the potential for greatly improved user convenience, the device currently has the following limitations, which also provide directions for improvement. Firstly, the system proposed in this study was not evaluated among people with a range of physical and physiological characteristics. Therefore, for generalizing the proposed system, a follow-up study is required that would include a group of subjects with characteristics that may affect the sensitivity of the sensor, such as varied body shapes and obesity. Secondly, this study only verified the QRS detection performance with the aim of evaluating the performance of the newly proposed ECG measurement system at an early stage; however, for clinical use in the future, the measurement performance of more sophisticated ECG waveforms, such as the PR interval, QT interval, corrected QT interval, and ST segment, should be verified. Thirdly, further verification through user satisfaction surveys are also required to verify the *user-friendliness* and *comfort* characteristics that are emphasized as strengths in this study. Technically, in the case of a clothing-type ECG measurement technology using textiles, the electrode is vulnerable to motion artifacts because its contact with skin does not use an adhesive. Moreover, the electrode used in this study was a dry electrode that does not use gels, and the measured signal quality can be greatly affected by individual characteristics, such as skin condition. In addition, the durability against washing, a typical problem of textile-based electrodes, has not been considered in this study; therefore, it will be necessary to contemplate approaches such as conductive and hydrophobic coatings in the future.

Some sampling was delayed or lost during the wireless data transmission, and it has been confirmed that data delay or loss usually occurs due to the Bluetooth communication. The cause of this phenomenon is not clear, but we presume it may be due to a communication channel problem, the Bluetooth version used, or the Bluetooth strategy of the smartphone. Optimizing the amount of transmitted data should be prioritized to reduce such transmission errors, and it is thought that performance can be improved by optimizing the sampling rate or compression transmission. Even if data loss is minimized, a transmission error occurring in the vicinity of QRS can lead to serious distortion of the waveform; therefore, research on postprocessing to compensate for this loss is also necessary. From the point of view of practical use, battery life is an important feature, but optimization of battery life and power consumption was not considered in this study. Therefore, in order to commercialize or secure practical use clinically, it will be necessary to optimize the capacity of the battery used and the power consumption of the system. In this process, a low-power MCU or a low-power design based on an interrupt in firmware can be applied. In addition, it is anticipated that battery life can be maximized through use scenarios in daily life, such as intermittent measurement.

ECGs measured with the system developed here were obtained with a modified limb lead, but this approach is known to exaggerate the amplitude of the P wave or the ST segment compared to the 12-lead standard ECG [21,28]. Therefore, it is difficult to envisage the modified limb approach replacing traditional standard limb leads, and the developed system is likely to be used for heart rate estimation and rhythmic arrhythmia detection; however, it cannot be generalized as being clinically applicable through waveform analysis. In addition, while the results of this study show that the system can be used for obtaining an ECG and calculating heart rate, it has not yet been verified on multiple subjects whose physiological characteristics may be quite diverse. Therefore, the stability and applicability of the system will need to be validated through further study on subjects exhibiting diverse individual characteristics that may vary according to criteria such as sex and age.

### Conclusions

In this study, we have developed a system that can measure an ECG and that can be worn in the same way as conventional clothing in terms of appearance and ease of use, without upper body compression; we also evaluated its feasibility as a heart rate monitor. The developed system was able to measure representative ECG waveform features without user awareness or reported discomfort; in addition, the system did a good job of tracking the heart rate, as confirmed independently by a commercial ECG monitoring device. The newly developed waistband-type, wearable, wireless ECG system is anticipated to be able to secure general usability through future research aimed at improving the reliability of wireless communication technology and optimizing power consumption. Since the proposed system has not yet been verified for users with various physical and physiological characteristics, it needs to be generalized through follow-up experiments involving more diverse subjects. Moreover, it will also be necessary to confirm the convenience of the proposed system through quantitative evaluation of user satisfaction. Finally, performance verification in terms of more detailed ECG waveform characteristic feature analysis is also required for expansion to a clinical ECG measurement system.

## Abbreviations

AV: atrioventricular
bpm: beats per minute
ECG: electrocardiogram
IRB: institutional review board
LoA: limits of agreement
MAE: mean absolute error
MAF: moving average filter
MAPE: mean absolute percentage error
MCU: microcontroller unit
RRI: RR interval
SA: sinoatrial
